# TNF/TNFR1 is a Key Regulator of Prolonged Fasting‐Induced Decrease in Adipose Tissue

**DOI:** 10.1096/fj.202501928RR

**Published:** 2026-01-08

**Authors:** Amanda Carla Clemente de Oliveira, Adma Maciel Babêtto, Mariele Lino Silva, Larisse de Souza Barbosa Lacerda, Thiago de Souza Rodrigues, Marina Chaves de Oliveira, Christine Rouault, Emmanuel L. Gautier, André Gustavo Oliveira, Karine Clément, Mauro Martins Teixeira, Geneviève Marcelin, Adaliene Versiani Matos Ferreira

**Affiliations:** ^1^ Immunometabolism, Department of Nutrition, Nursing School Federal University of Minas Gerais Belo Horizonte Brazil; ^2^ Drug Research and Development Center Federal University of Minas Gerais Belo Horizonte Brazil; ^3^ INSERM, Nutrition and Obesities: Systemic Approach Research Group, Nutriomics Sorbonne University Paris France; ^4^ Computer Department Federal Center for Technological Education of Minas Gerais Belo Horizonte Brazil; ^5^ Department of Physiology and Biophysics Federal University of Minas Gerais Belo Horizonte Brazil

**Keywords:** adipose tissue, bariatric surgery, Interleukin‐18, prolonged fasting, tumor necrosis factor

## Abstract

Nutrient availability influences white adipose tissue (WAT) inflammation, leading to decreased or increased adiposity. Tumor necrosis factor (TNF) is elevated in WAT under both conditions, being involved in lipolysis and regulation of interleukin 18 (IL‐18) secretion, which may regulate lipolytic processes. However, the role of these cytokines in adiposity reduction due to low energy availability remains unclear. Wild‐type (WT) mice fasted for 24 h showed decreased adiposity, whereas TNFR1 knockout mice (TNFR1^−/−^) were unresponsive to fat pad loss, even after 48‐h fasting. TNFR1^−/−^ mice were also resistant to *β*3‐adrenergic receptor agonist CL316,243‐induced fat mobilization, which was linked with reduced expression of lipases, *β*3‐adrenergic receptors, and cytokines in WAT. Also, mice treated with the TNF‐α inhibitor infliximab and fasted for 48 h showed resistance to adiposity loss, suggesting that prolonged fasting‐induced TNF signaling may modulate adipose tissue reduction. Conversely, IL‐18 does not seem to influence fat pad loss induced by 24‐h fasting as IL‐18 knockout mice (IL‐18^−/−^) express TNF in WAT and respond to prolonged fasting similarly to WT animals. To assess the potential translational relevance of our findings to human obesity, we analyzed 53 samples from patients with obesity who underwent bariatric surgery. Interestingly, TNFR1 and IL‐18 expressions in sWAT correlate with the expression of lipases and adipokines in the subcutaneous site despite no correlation with body weight or fat mass 1 year after surgery. In summary, this study suggests that the TNF/TNFR1 axis is crucial for metabolic adaptation and is a prerequisite for prolonged fasting‐induced lipolysis in male mice.

## Graphical abstract

Fasting triggers an inflammatory response and increases sympathetic tone in white adipose tissue (WAT), promoting fat breakdown and tissue remodeling. Fasting sparks an inflammatory response that underpins the reduction in adipocyte size and WAT remodeling. The cytokine TNF is fundamental for this remodeling, as its absence attenuates fat loss. Conversely, IL‐18 does not seem to exert a significant influence; its loss even promotes appropriate remodeling by increasing TNF expression. TNF/TNFR1 is, therefore, essential in fasting‐induced adiposity loss.

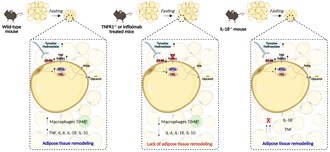

## 1 Introduction

The white adipose tissue (WAT) is the primary organ responsible for energy storage and disposal [1]. Consequently, WAT can remodel itself according to the body's energy status. During periods of low energy availability, such as prolonged fasting, dietary calorie restriction, and after bariatric surgery, adipose tissue is reduced to provide energy substrate, thereby protecting the organism from hypoglycemia. A slight fluctuation in blood glucose levels increases the sympathetic tone in the hypothalamus, which in turn leads to the secretion of noradrenaline in the adipose tissue [2]. This neurotransmitter interacts with the *β*3‐adrenergic receptor (*β*3‐AR), initiating a signaling cascade that activates lipolysis pathways. These pathways involve key lipases, including adipose triglyceride lipase (ATGL) and hormone‐sensitive lipase (HSL). As a result, triglycerides stored in adipocytes are broken down into free fatty acids (FFA) and glycerol, which are then utilized as energy sources by other organs, including muscles and the liver [3].

In the last few years, it was suggested that inflammation may support fatty acid release from WAT during low‐energy availability situations, especially for prolonged periods. For instance, macrophages infiltrate the visceral adipose tissue in fasting mice [4]. Akin to this, lean mice exhibit higher expression of acute‐phase proteins in their inguinal adipose tissue (iWAT) after 24 h of fasting [5]. The epididymal adipose tissue (eWAT) of fasted mice also shows an increase in neutrophil numbers and higher levels of TNF, IL‐6, IL‐10, and TGF‐*β* compared to fed mice [6]. Ten days of fasting in humans lead to higher serum levels of TNF, IL‐6, and IL‐10 and an increase in macrophage numbers in the subcutaneous adipose tissue [7]. Targeting *β*3‐AR signaling using CL316,243 also induces a heightened inflammatory response in the adipose tissue of mice [8, 9, 10]. Accordingly, the reduction of adipose tissue induced by prolonged fasting is compromised in a mouse model of inflammatory hyporesponsiveness [11]. Interestingly, mice with obesity who were subjected to 24‐h fasting resist adipose tissue reduction. This phenomenon can be linked to a dysfunctional inflammatory response to prolonged fasting, which we termed “inflammatory inflexibility” in obesity [6].

In adipocytes, TNF signaling via TNFR1 induces lipolysis [12] by inhibiting Gi protein and activating NF‐κB and MAPK pathways [13, 14, 15, 16]. TNF also stimulates inflammasome activation and IL‐18 secretion [17]. Additionally, IL‐18 hyperexpression in mice has been associated with increased fat catabolism [18]. This evidence suggests that inflammatory signaling contributes to fat pad reduction. Therefore, our study aims to elucidate the TNFR1‐dependent molecular mechanism regulating lipolysis and white adipose tissue remodeling induced by fasting, primarily utilizing non‐obese male mice as the main model. We demonstrated a cytokine‐specific effect in prolonged fasting‐induced adiposity loss using knockout and pharmacological approaches in animal models. Our findings highlight TNF/TNFR1 as a key mediator of adipose tissue remodeling, whereas IL‐18 has a minimal impact.

## 2 Material and Methods

### 2.1 Experimental Design

The Ethics Committee approved all experimental procedures for the Use of Animals of UFMG (protocol numbers 16/2020 and 22/2023) following the ARRIVE guidelines. The mice were between 24 and 30 weeks old and housed in groups at room temperature, 28°C, with a 12‐h light/dark cycle (7:00–19:00). Male C57Bl/6J mice (WT) and male TNFR1^−/−^ and IL‐18^−/−^ mice (background C57Bl/6J) were obtained from the University's Animal Facility and the Laboratory of Gnotobiology and Immunology situated at UFMG (*n* = 48). The WT mice were not littermate controls for the knockout strains, but all mice were maintained on a C57Bl/6J genetic background and matched by age and initial weight to ensure consistency. A set of WT, TNFR1^−/−^, and IL‐18^−/−^ mice (*n* = 8 in each group) were used in the prolonged fasting experiments, which started between the second and third hours of the light cycle (ZT2–ZT3) and were finished after 24 h or 48 h, according to each experiment. The diet was removed for 3 h for the control groups, starting from the first hour of the light cycle (ZT1). We also treated C57Bl/6J mice (*n* = 30) with an intraperitoneal injection of infliximab (Merk, Darmstadt, Germany, Y0002047) at a dose of 10 mg/kg or PBS in equivalent volume between the first and the third hour of the light cycle (ZT1–ZT3). Subsequently, mice were fasted for 48 h. At 24 h of fasting, mice received another shot of infliximab or PBS. As in the fasting protocol, the diet was removed from the control group, and the animals were given an injection of PBS (*n* = 5–8 in each group). During the experiments, the animals had free access to water. Another set of WT and TNFR1^−/−^ animals (*n* = 30) received an intraperitoneal injection of CL316,243 at a dose of 1 mg/kg or a corresponding volume of PBS between the second and third hours of the light cycle (ZT2–ZT3) (*n* = 7–8 in each group). In the experiments cited herein, they had free access to water and food and were euthanized after 24 h. During all fasting experiments, animals were provided with free access to water, environmental enrichment, and cages containing wood shavings to minimize potential distress.

### 2.2 Human

Patients with severe obesity (BMI ≥ 40 kg/m^2^) from the BARICAN cohort were recruited and accompanied by the Nutrition Department of Pitié‐Salpêtrière Hospital (Paris, France). The clinical and anthropometric characteristics of the study participants are described in Table S1. This cohort is approved by CNIL (Commission Nationale de l'Informatique et des Libertés; No. 1222666) as by French Research Ministry. The patients signed the informed consent form to be part of a range of studies registered in the clinical trials platform (https://clinicaltrials.gov P050318 Les Comités de Protéction des Personnes (CPP) approval: November 24, 2006, NCT01655017, NCT01454232). All the patients complied with standard recommendations for bariatric surgery and were monitored according to French and international guidelines.

Samples from omental (oWAT) and subcutaneous (sWAT) adipose tissues were collected during bariatric surgery and stored in a −80°C freezer. The anthropometric data were measured by the whole‐body dual‐energy X‐ray absorptiometry (DEXA) technique with a fan beam (Hologic Discovery W software, version 12.6; Hologic Inc). The body weight loss, fat mass loss, android fat loss, and gynoid fat loss were calculated using the dates before and 1 year after bariatric surgery. Blood was collected after a 12‐h overnight fast, and the serum was analyzed for blood glucose, insulinemia, and glycated hemoglobin (HbA1c) using colorimetric assays.

### 2.3 Ex Vivo

Portions of eWAT and iWAT from C57BL/6J mice were weighed, harvested, and incubated with DMEM without red phenol. First, the samples were treated with 2 μg/mL of infliximab [19] (Merk, Darmstadt, Germany, Y0002047) or PBS in equal volume for 24 h. The samples were stimulated or not with CL316,243 at a concentration of 10 μM for 90 min. Glycerol was measured in the medium with a Glycerol assay kit (Sigma Aldrich, San Luis, Missouri, USA, MAK117), and qPCR was performed in the tissue.

### 2.4 Serum Biochemistry

Serum concentrations of FFA (Wako Pure Industries, Osaka, Japan) and leptin (R&D Systems Europe Ltd., Abington, UK) were determined respectively by colorimetric method and enzyme‐linked immunosorbent assay (ELISA).

### 2.5 RNA Extraction, cDNA Synthesis, and Quantitative Polymerase Chain Reaction (qPCR)

RNA from eWAT and iWAT was extracted with the Pure Link RNA Mini Kit (ThermoFisher, Massachusetts, USA), and RNA from oWAT and sWAT was extracted with the RNeasy Micro Kit (Qiagen, Hilden, Germany). All the samples were synthesized with the cDNA synthesis kit (Reverse Transcription System; Promega, Madison, WI, USA). Then, the qPCR was performed with SYBR Green PCR Master Mix (ThermoFisher, Massachusetts, EUA) in the Step One machine (ThermoFisher, Massachusetts, EUA). Relative levels of gene expression were determined by the 2^−∆∆Ct^ method. The primers used are described in Table S2.

### 2.6 Histomorphometry

Portions of eWAT and iWAT were fixed in 10% formaldehyde for 48 h and then kept in 70% ethanol for 24 h. After this, the samples underwent dehydration and diaphanization before being embedded in paraffin. Blocks were then cut into 5‐μm sections and stained with Hematoxylin–Eosin (H&E). Images from six fields of the eWAT and iWAT of each animal were captured using a digital camera attached to a microscope (200×). The area of 50 cells was measured in each mouse using the precise ImageJ software (National Institute of Health, Bethesda, Maryland, USA) to calculate the area of each adipocyte (μm^2^). The average adipocyte area of each animal was then calculated, ensuring an accurate analysis.

### 2.7 Immunofluorescence

The eWAT and iWAT sections embedded in paraffin were deparaffinized and hydrated. Then, the samples were submitted to antigen retrieval with a citrate buffer. Immunofluorescence was performed with a Tyramide SuperBoost Alexa 647 kit (Invitrogen, Waltham, Massachusetts, USA). The primary antibodies used were tyrosine hydroxylase (OPA104050) and *β*3‐AR (PA550914) (Invitrogen, Waltham, Massachusetts, USA), and the secondary antibody was anti‐rabbit (BA‐1400, Vector Labs, Malvern, USA). All the antibodies were diluted at 1:200. Slides were mounted with Fluoromount‐G with DAPI (00–4959‐52, Invitrogen, Waltham, Massachusetts, USA). Images of 5 fields per sample were taken in the microscope Apotome 2 (Zeiss) from the Image Acquisition and Processing Center of UFMG. The images were analyzed using ImageJ software (National Institute of Health, Bethesda, Maryland, USA).

### 2.8 Flow Cytometry

Portions of eWAT and iWAT were harvested and digested in a solution containing DMEM, bovine serum albumin (1%), HEPES (10 mM), and collagenase A (1 mg/mL, Roche) for 1 h at 37°C under agitation. The tissue was washed in a PBS buffer containing bovine serum albumin (1%) and EDTA (0.5%). The adipocytes were separated from the stroma vascular fraction and discarded with a vacuum pump. Then, the stroma vascular fraction was centrifuged and passed through a 100‐μm filter before staining. The antibodies used are from BD Biosciences: CD45, Singlec F, CD64, and TIM4. Data were acquired on a BD LSRFortessa flow cytometer (BD Biosciences, USA) and analyzed with FlowJo software (Tree Star).

### 2.9 Analysis of Published Single‐Nucleus RNA Sequencing Data

Analysis of published RNA Sequencing Data Expression of TNF was performed from the dataset obtained from Emont et al. [20] using the R package Seurat [21, 22].

### 2.10 Statistics

The groups were divided randomly, and the mean body weight was statistically not different for each experimental group. We used Kolmogorov–Smirnov to analyze the normality of all data. All Statistical comparisons for parametric samples between the various groups were performed by ANOVA “two‐way” with Tukey's post hoc test. At the same time, the Student's t‐test was used to compare two groups. To compare the different groups, the Kruskal–Wallis test was performed for nonparametric samples. For comparisons between two nonparametric groups, the Mann–Whitney test was performed. Results are presented as the mean ± SEM. We used GraphPad 9 Software Inc. (San Diego, CA, USA) to conduct all analyses and assemble the graphs in the mouse experiments. We calculated the sample size with GPower Software (version 3.1.9.7) with an ANOVA two‐way test. The parameters were effect size = 0.8, α error = 0.05, and statistical power = 0.9, and the sample size was 32 animals = 5 mice in each group. A blind investigator performed the immunofluorescence. Grubbs' test determined outliers between the samples. Values considered unusual and, therefore, excluded from analyses were statistically lower than 0.05. Spearman's correlation was performed with the R package Hmisc [23], and matrix correlation was made with the R package Corrplot [24].

## 3 Results

### 3.1 TNFR1 Is Essential for Effective Adipose Tissue Reduction in Mice

**FIGURE 1:**
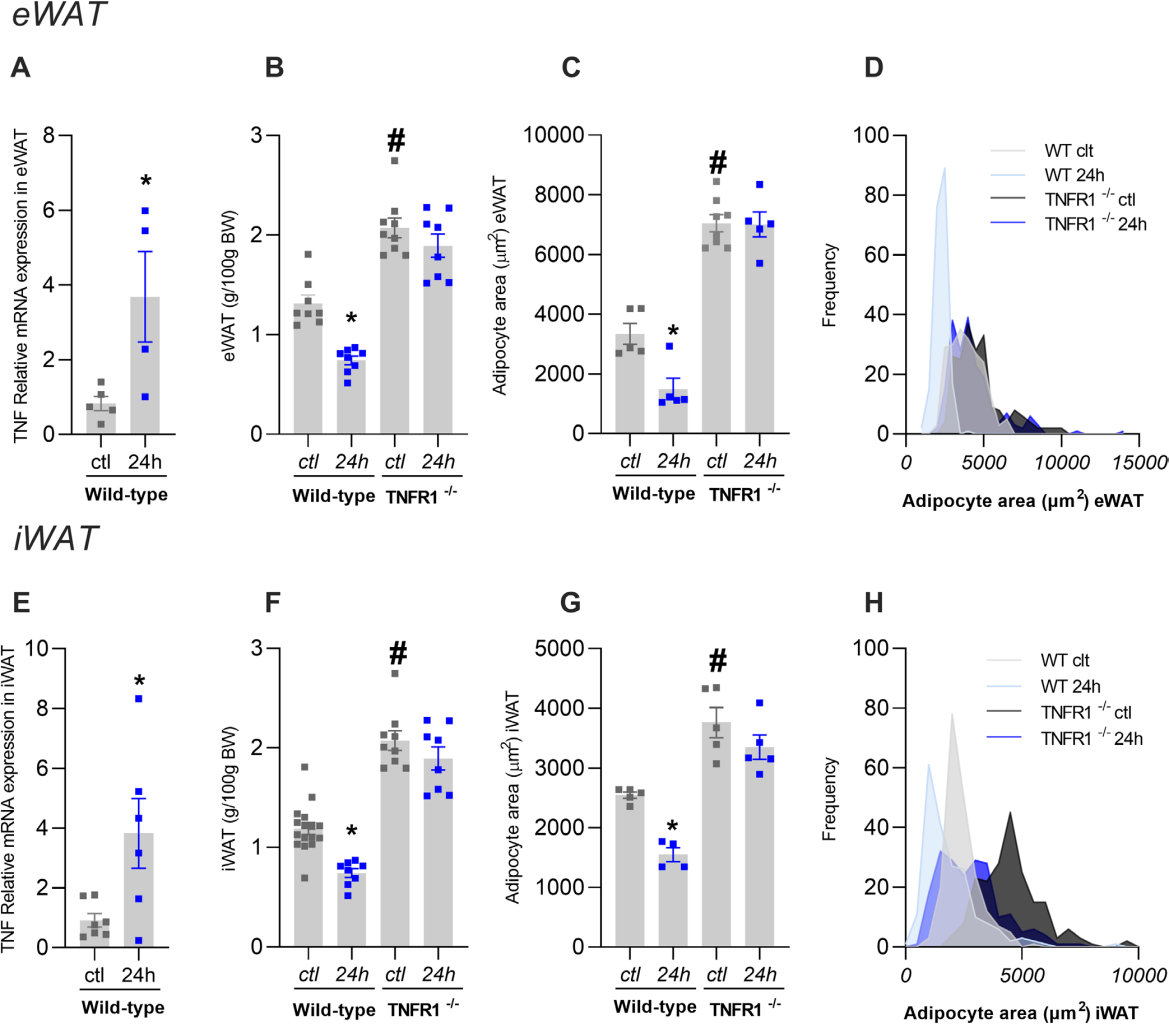
TNFR1 participates in adipose tissue reduction. Wild‐type (WT) or TNFR1^−/−^ mice were submitted (24‐h) or not (ctl) to 24‐h fasting. (A) TNF expression in eWAT from WT mice. B to D is referred to analysis in eWAT, which includes (B) weight, (C) adipocyte area, and (D) adipocyte area distribution. TNF expression in iWAT from WT mice (E). F to H is referred to analysis in iWAT, which includes (F) weight, (G) adipocyte area, and (H) adipocyte area distribution. The frequency distribution was generated from the area of all evaluated adipocytes per experimental group (eWAT: *N* = 200; iWAT: *N* = 200), using a bin range (eWAT: 14000 μm^2^; iWAT: 10000 μm^2^). The mean adipocyte area for each animal and the variance between groups are represented in C and G. *n* = 6–9, *p < 0.05 vs. respective ctl group, #*p* < 0.05 vs. wild‐type in respective treatment, two‐way ANOVA, Tukey's post hoc test; Student's T‐test between two groups.

Wild‐type mice subjected to 24‐h fasting exhibited increased TNF expression in both epididymal (eWAT) and inguinal white adipose tissue (iWAT) (Figure 1A,E). Since TNF primarily signals through the receptor superfamily TNFRSF1, including TNFR1 and TNFR2, we analyzed the expression of these receptors in adipocytes from both mice and humans using the snRNAseq data from Emont et al. [20]. Our analysis revealed that TNFR1 is more predominantly expressed than TNFR2 in adipocytes, with 13.02% of mouse adipocytes expressing TNFR1 compared to 8.11% for TNFR2 (*p* < 2.2e–16, Figure S1A). In humans, the disparity is even greater, with 24.16% of adipocytes expressing TNFR1 versus 2.63% expressing TNFR2 (*p* < 2.2e–16, Figure S1B). Importantly, the mAd3 cluster exhibits significantly higher TNFR1 expression (0.531) than TNFR2 (0.113), with a *p*‐value of 3.34e‐23. This pronounced disparity highlights that TNFR1 expression is substantially enriched in the mAd3 cluster relative to TNFR2, underscoring the specificity and predominance of TNFR1 in this lipolytic adipocyte subpopulation, indicating a significant enrichment of TNFR1 in these cells Table S3 and Figure S2C. Therefore, to further investigate the role of TNF in adipose tissue fat loss, we used a mouse model lacking Tnfr1, the primary receptor mediating TNF signaling in adipocytes (TNFR1^−/−^). Following 24‐h fasting, WT mice exhibited significant eWAT and iWAT mass reductions, along with smaller adipocytes (Figure 1B,C; F–G). In contrast, TNFR1^−/−^ mice were unresponsive to prolonged fasting‐induced reduction in eWAT and iWAT masses and adipocyte areas (Figure 1B,C; F–G).

**FIGURE 2:**
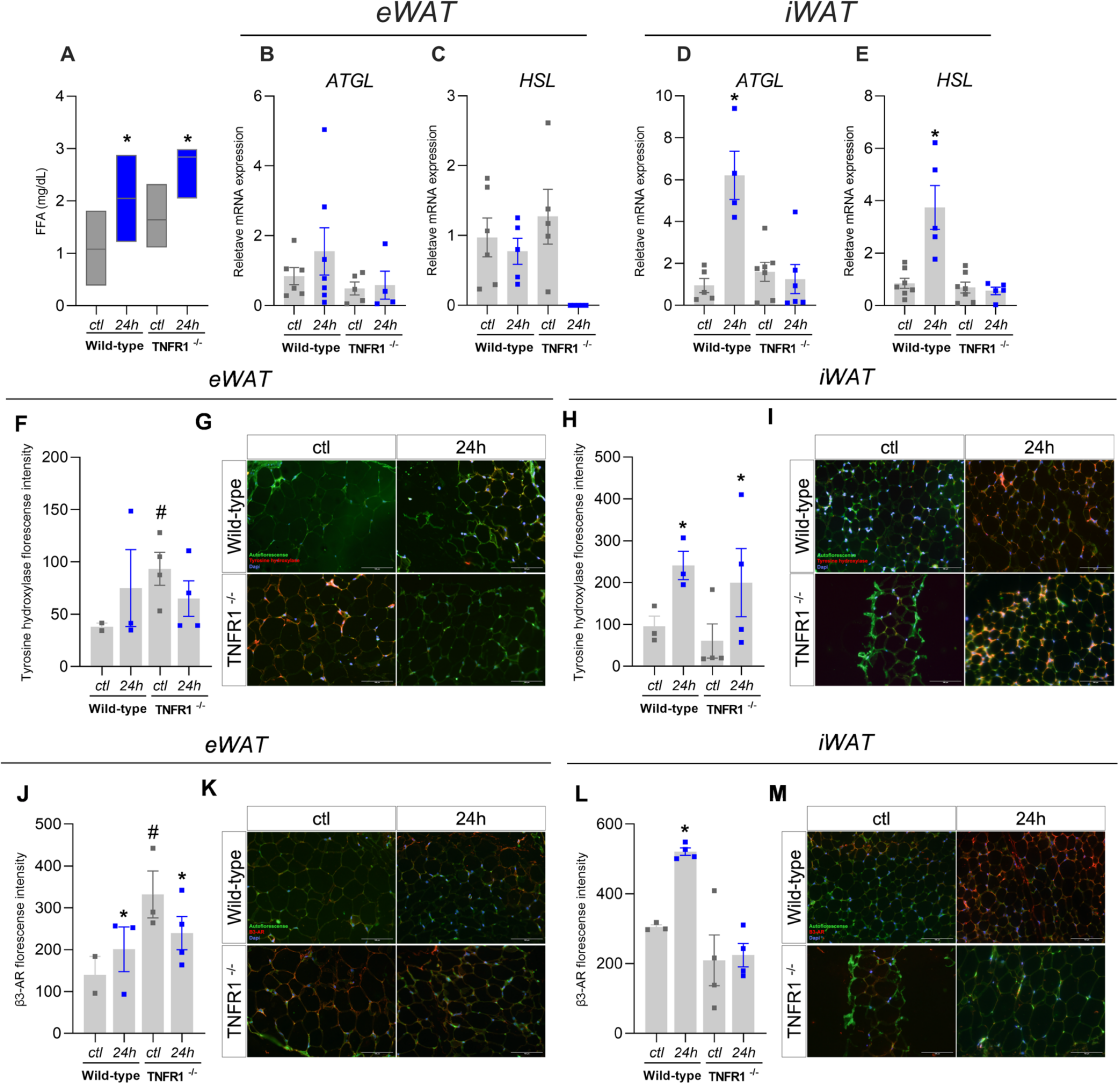
TNFR1 modulates the expression of lipolysis markers and cytokines in adipose tissue from mice under prolonged fasting. Wild‐type or TNFR1^−/−^ mice were submitted (24‐h) or not (ctl) to 24‐h fasting. (A) Serum FFA levels, ATGL and HSL expression in (B and C) eWAT and (D and E) iWAT, *n* = 4–8. F‐I refers to tyrosine hydroxylase analysis, which includes (F) fluorescence intensity in eWAT, (G) eWAT representative images, (H) fluorescence intensity in iWAT, and (I) iWAT representative images. J–M refers to the *β*3‐AR analysis, which includes (J) fluorescence intensity in eWAT, (K) eWAT representative images, (L) fluorescence intensity in iWAT, and (M) iWAT representative images. *n* = 2–4, *p < 0.05 vs. respective ctl group, #*p* < 0.05 vs. wild‐type in respective treatment, two‐way ANOVA, Tukey's post hoc test.

While prolonged fasting increased serum FFA levels in both wild‐type and TNFR1^−/−^ mice (Figure 2A), the mRNA expression of key adipocyte lipases, ATGL and HSL in eWAT, remained unchanged between baseline and 24‐h fasting conditions (Figure 2B,C). By contrast, in iWAT, the lipase expression increased in WT mice but not in TNFR1^−/−^ mice following prolonged fasting (Figure 2D,E). The distinct response of lipolytic enzymes in eWAT and iWAT following fasting can be explained, at least in part, by the difference in TNFR1 expression between these fat depots. Indeed, iWAT expresses 70% more TNFR1 than eWAT Figure S2A,B, suggesting that iWAT may be more sensitive to TNFR1 deficiency than eWAT.

Given the crucial role of sympathetic innervation in regulating fat pad loss during prolonged fasting [2], we conducted an immunofluorescence analysis for tyrosine hydroxylase (TH) and *β*3‐adrenergic receptor (*β*3‐AR). The epididymal adipose tissue from mice lacking TNFR1 showed higher fluorescence intensity for TH than WT despite prolonged fasting not changing the TH staining levels (Figure 2F,G). In iWAT, 24‐h fasting induced a higher fluorescence intensity of TH in wild‐type and TNFR1^−/−^ mice (Figure 2H,I), suggesting that prolonged fasting induces an increase in TH content to support the local production of catecholamines that, in turn, can mediate the lipolysis rate. We further analyzed the *β*3‐AR content in adipose tissue. Fasting increased the *β*3‐AR content in eWAT and iWAT from WT mice, an effect not seen in TNFR1^−/−^ mice (Figure 2J–M). These data indicate that TNFR1 deficiency may alter prolonged fasting, mediating catecholamine signaling by limiting *β*3‐AR content in adipocytes.

**FIGURE 3:**
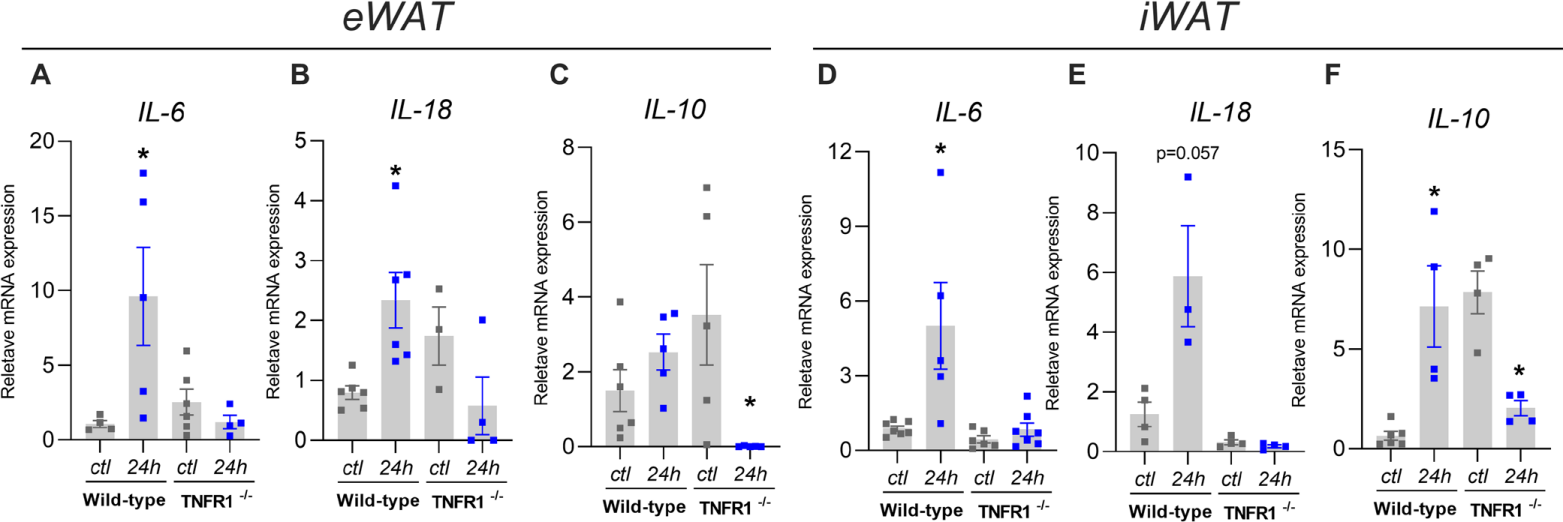
TNFR1 modulates cytokine expression in fat pad loss induced by prolonged fasting. Wild‐type or TNFR1^−/−^ mice were submitted (24‐h) or not (ctl) to 24‐h fasting. (A) IL‐6, (B) IL‐18, (C) IL‐10 expression in eWAT and (D) IL‐6, (E) IL‐18, (F) IL‐10 expression in iWAT. *n* = 4–8 *p < 0.05 vs. respective ctl group, two‐way ANOVA, Tukey's post hoc test.

To further investigate the underlying mechanism, we analyzed the expression of cytokines known to be induced by TNFR signaling. Our data show a significant increase in several cytokines following prolonged fasting. Specifically, WT mice subjected to a 24‐h fast exhibited a marked increase in the expression of IL‐6 (Figure 3A,D), IL‐18 (Figure 3B,E), and IL‐10 (Figure 3C,F) in both epididymal and subcutaneous adipose tissue. In contrast, the expression of these cytokines in the adipose tissue of TNFR1^−/−^ mice remained unchanged after fasting, with IL‐10 levels notably reduced (Figure 3A–F). Thus, the lack of TNF signaling appears to impair both fat pad reduction and the cytokine response typically triggered by 24‐h fasting.

**FIGURE 4:**
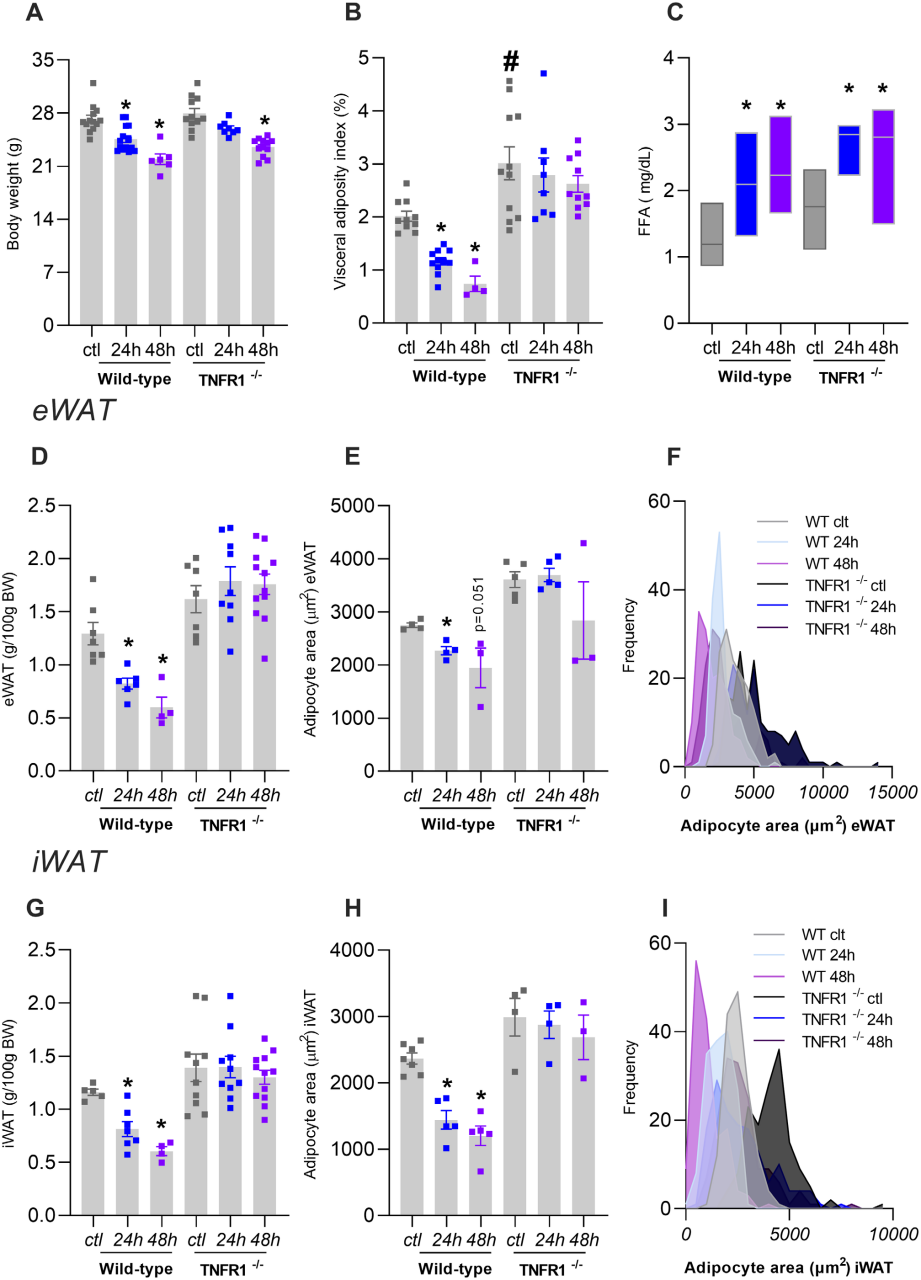
TNFR1^−/−^ mice resist adipose tissue reduction induced by 48 h of fasting. Wild‐type or TNFR1^−/−^ mice were submitted or not (ctl) to 24‐h or 48‐h fasting. (A) Body weight, (B) adiposity index (the sum of epididymal, mesenteric, and retroperitoneal WAT expressed as a percentage of body weight), (C) serum FFA, (D) eWAT weight, (E) eWAT adipocyte area, (F) eWAT adipocyte area distribution, (G) iWAT weight, (H) iWAT adipocyte area, (I) iWAT adipocyte area distribution. The frequency distribution was generated from the area of all evaluated adipocytes per experimental group (eWAT: *N* = 150; iWAT: *N* = 150), using a bin range (eWAT: 14000 μm^2^; iWAT: 9500 μm^2^). The mean adipocyte area for each animal and the variance between groups are represented in E and H. *n* = 4–8, *p < 0.05 vs. respective ctl group, #*p* < 0.05 vs. wild‐type in respective treatment, two‐way ANOVA, Tukey's posthoc test.

We next investigated whether the lack of response to 24‐h fasting observed in TNFR1−/− mice persisted after an even more prolonged fasting period. After 24 or 48 h of fasting, both WT and TNFR1^−/−^ mice exhibited reduced body weight (Figure 4A). In WT mice, 48 h of fasting led to further visceral adiposity loss (Figure 4B), whereas TNFR1−/− mice remained unresponsive to fat mass reduction even after 48‐h fasting. Although fasting increased circulating FFA levels in both groups (Figure 4C), only WT mice showed a significant reduction in fat pad mass and smaller adipocytes in both eWAT (Figure 4D–F) and iWAT (Figure 4G–I) compared to TNFR1^−/−^ mice.

### 3.2 Infliximab Blocks eWAT and iWAT Mass Reduction Induced by Prolonged Fasting in Mice

**FIGURE 5:**
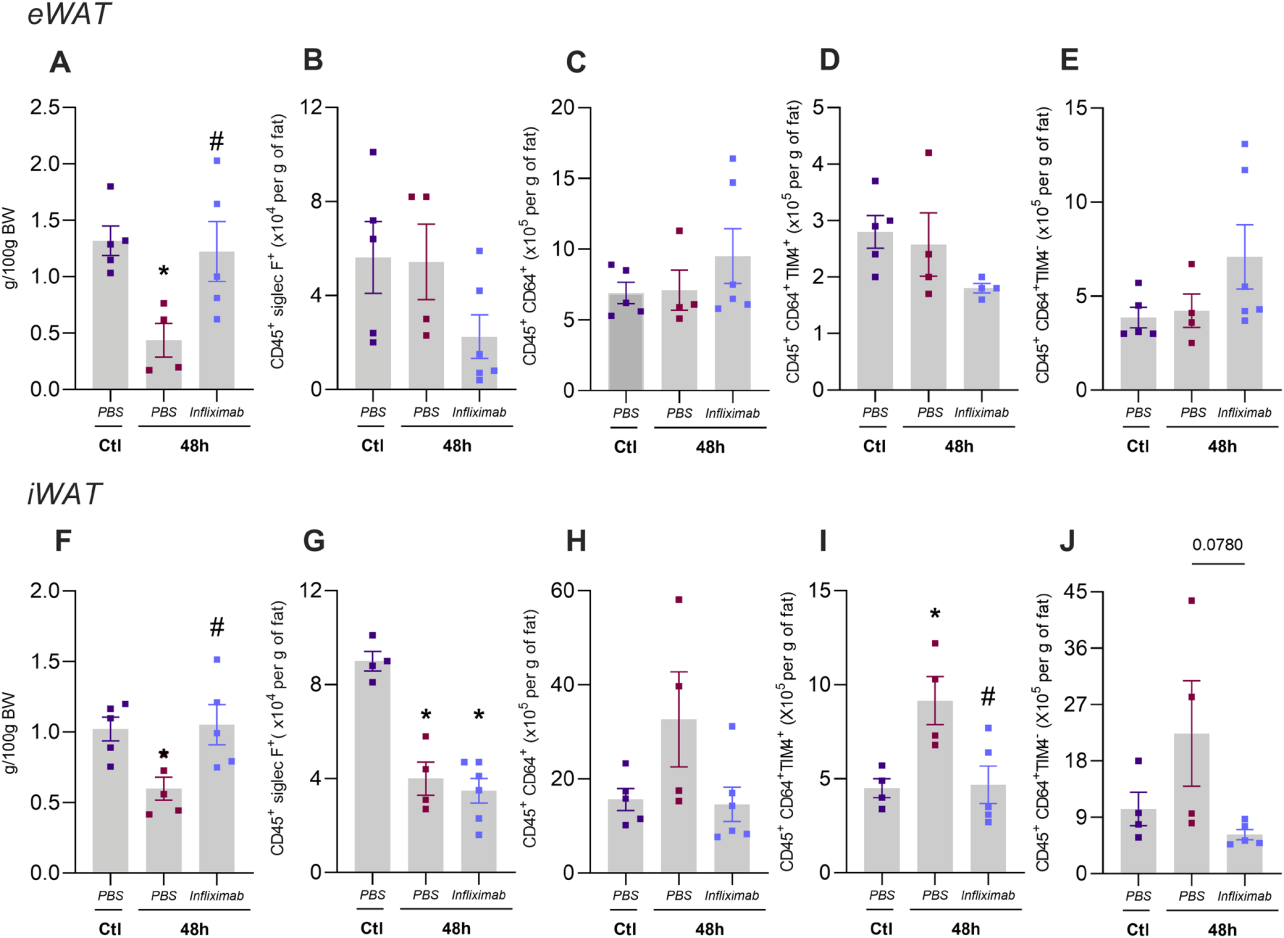
Anti‐TNF treatment inhibits adipose tissue reduction and macrophage number in WAT. Wild‐type mice were treated with PBS or infliximab and submitted (48‐h) or not (ctl) to 48‐h fasting. A to E is referred to analysis in eWAT, which includes (A) weight, (B) eosinophils, (C) total macrophages, (D) resident macrophages, and (E) recruited macrophages. F to J is referred to analysis in iWAT, which includes (F) weight, (G) eosinophils, (H) total macrophages, (I) resident macrophages, and (J) recruited macrophages. *n* = 4–8, p < 0.05 vs. respective ctl group, #p < 0.05 vs. wild‐type in respective treatment, two‐way ANOVA, Tukey's post hoc test.

To validate our findings in TNFR1^−/−^ mice, we examined whether similar results could be observed in mice treated with infliximab, a TNF‐inhibiting antibody [26]. Wild‐type mice were treated with infliximab or vehicle prior to fasting, and after 24 h, the animals underwent a 48‐h fasting period. Like TNFR1^−/−^ mice, infliximab‐treated mice showed no significant change in eWAT and iWAT mass compared to the vehicle group (Figure 5A,F). Nevertheless, no significant alterations in lipase expressions were detected among the experimental groups Figure S3A–D.

Given that immune cells infiltrating adipose depots may respond to both prolonged fasting and the loss of TNF signaling, we further investigated the leukocyte population, including eosinophils and macrophages [4]. The gate strategy is available in Figure S3E. In eWAT, neither prolonged fasting nor infliximab treatment altered the population of eosinophils (CD45^+^ siglecF^+^), resident macrophages (CD45^+^ CD64^+^ TIM4+), or recruited macrophages (CD45^+^ CD64^+^ TIM4^−^) (Figure 5B–E). However, prolonged fasting led to a reduction in eosinophils (Figure 5G) in iWAT, an effect that was unaffected by infliximab treatment. Additionally, iWAT showed a trend toward an increased total macrophage count (Figure 5H), which included resident (CD45^+^ CD64^+^ TIM4^+^) (Figure 5I) and recruited macrophages (CD45^+^ CD64^+^ TIM4^−^). This increase was not shown in the infliximab‐treated mice (Figure 5J). Therefore, these findings suggest that TNF signaling plays a role in mediating prolonged fasting‐induced macrophage infiltration.

### 3.3 TNF Pathway Participates in Adipose Tissue Reduction Induced by CL316,243

The findings obtained from our prolonged fasting experiments support the role of the TNF pathway in adipose tissue reduction. Based on this, we investigated whether TNF signaling also mediates the fat pad loss induced by targeting β3‐AR with CL316,243. To test this, we stimulated explants from epididymal and inguinal adipose tissue of mice with CL316,243 in the presence or absence of infliximab in vitro. β3‐AR activation significantly increased glycerol release from adipocytes, indicating a substantial rise in lipolysis rate Figure S4A,F. Moreover, CL316,243 stimulation elevated the expression of TNF and IL‐6 in both eWAT and iWAT, while no change was observed in IL‐18 or IL‐10 expression Figure S4B–E,G–J. Infliximab effectively suppressed CL316,243‐stimulated glycerol release, but only in iWAT explants Figure S4A,F. Furthermore, the anti‐TNF treatment inhibited CL316,243‐induced increase in TNF expression in both eWAT and iWAT explants Figure S4B,G. No significant changes were observed in IL‐6, IL‐18, or IL‐10 expression in either inguinal or epididymal explants treated with infliximab Figure S4C–E,H–J.

**FIGURE 6:**
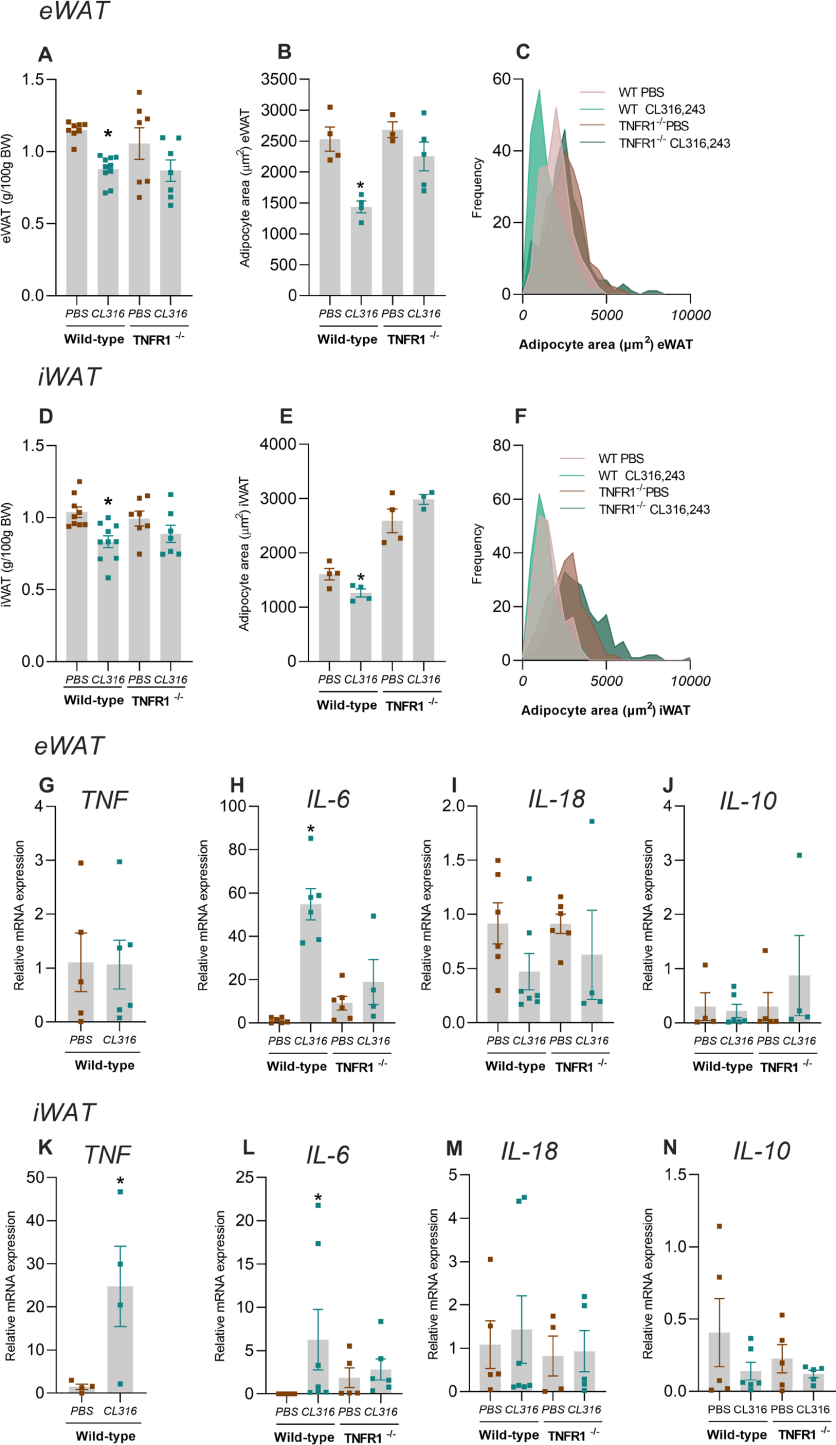
TNFR1 participates in WAT reduction induced by CL316,243 in mice. Wild‐type or TNFR1^−/−^ mice were treated with PBS or CL316,243. (A) eWAT weight, (B) eWAT adipocyte area, (C) eWAT adipocyte area distribution, (D) iWAT weight, (E) iWAT adipocyte area, (F) iWAT adipocyte area distribution. The frequency distribution was generated from the area of all evaluated adipocytes per experimental group (eWAT: *N* = 200; iWAT: *N* = 200), using a bin range (eWAT: 8500 μm^2^; iWAT: 10000 μm^2^). The mean adipocyte area for each animal and the variance between groups are represented in B and E. Expression of TNF (G), IL‐6 (H), IL‐18 (I), and IL‐10 (J) in eWAT, expression of TNF (K), IL‐6 (L), IL‐18 (M), and IL‐10 (N) in iWAT, *n* = 4–8, *p < 0.05 statistical difference between PBS vs. CL316,243, two‐way ANOVA, Tukey's post hoc test; Student's t‐test between two groups.

In vivo administration of CL316,243 to WT animals significantly reduced eWAT and iWAT fat pad mass, decreased adipocyte area, and increased the proportion of smaller adipocytes (Figure 6A–F). In contrast, TNFR1^−/−^ mice resisted the reduction in eWAT and iWAT following CL316,243 treatment (Figure 6A–F). WT mice treated with the *β*3‐AR agonist exhibited increased TNF expression in iWAT, while no change was observed in eWAT (Figure 6G,K). Moreover, CL316,243 administration led to elevated IL‐6 expression in both eWAT and iWAT of WT mice (Figure 6H,L). However, in TNFR1^−/−^ mice, IL‐6 expression in both eWAT and iWAT remained unchanged after CL316,243 treatment (Figure 6H,L). No significant change was observed in IL‐18 or IL‐10 expression in WAT of either WT or TNFR1^−/−^ mice (Figure 6I,J; M–N). Akin to what was found in the prolonged fasting challenge, the treatment with CL316,243 in mice lacking TNF signaling makes them resistant to adipose tissue reduction. The explant and in vivo data indicate that TNF is a critical cytokine in the lipolytic process induced by CL316,243, acting independently of other cytokines.

### 3.4 IL‐18 Does Not Regulate the Adipose Tissue Reduction Induced by Prolonged Fasting

**FIGURE 7:**
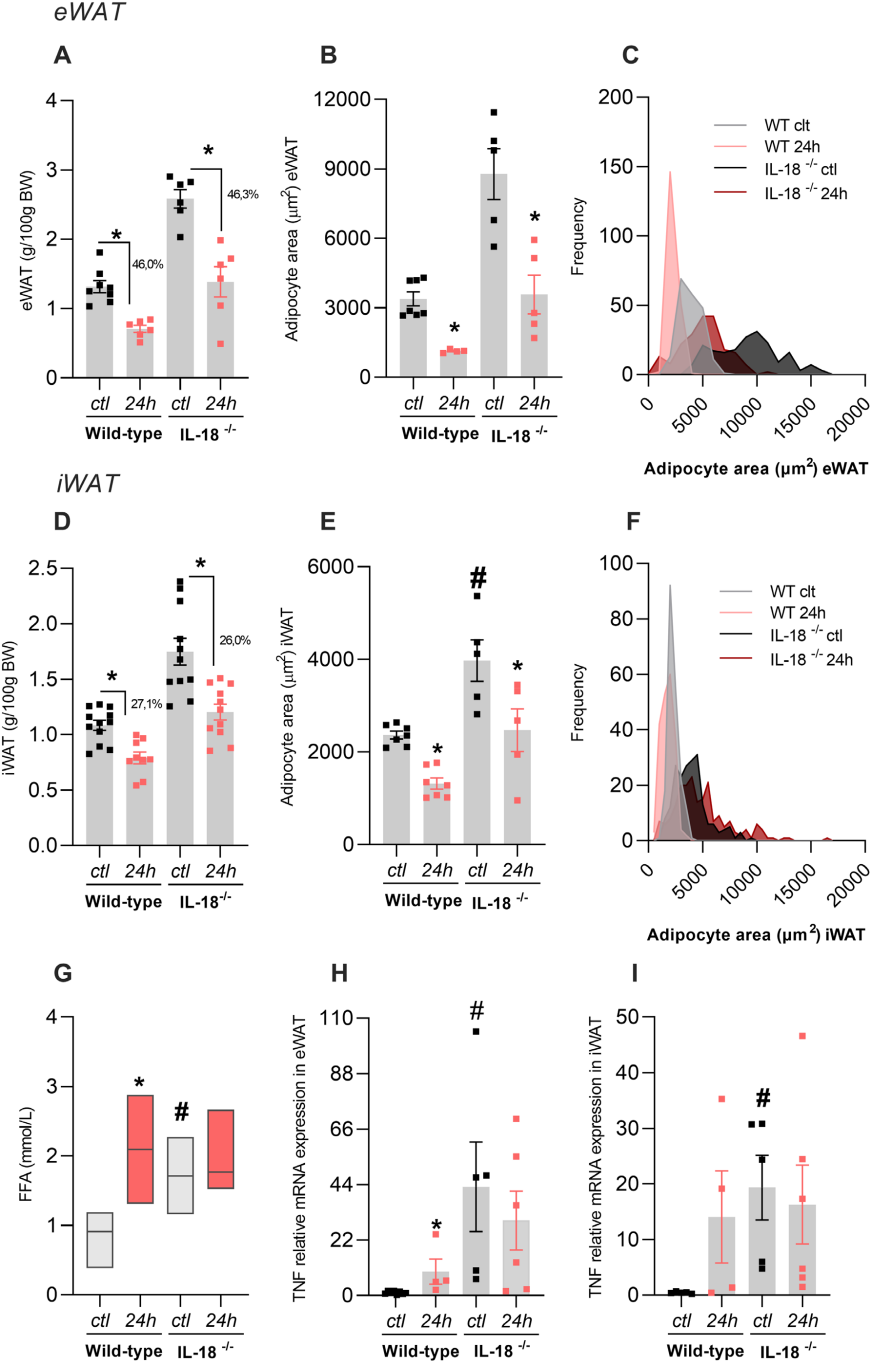
IL‐18 does not participate in adipose tissue reduction. Wild‐type or IL‐18−/− mice were submitted (24‐h) or not (ctl) to 24‐h fasting. (A) eWAT weight, (B) eWAT adipocyte area, (C) eWAT adipocyte area distribution, (D) iWAT weight, (E) iWAT adipocyte area, (F) iWAT adipocyte area distribution. The frequency distribution was generated from the area of all evaluated adipocytes per experimental group (eWAT: *N* = 200; iWAT: *N* = 200), using a bin range (eWAT: 23000 μm^2^; iWAT: 17000 μm^2^). The mean adipocyte area for each animal and the variance between groups are represented in B and E. (G) Serum FFA, TNF expression in (H) eWAT and (I) iWAT. *n* = 4–8, *p < 0.05 vs. respective ctl group, #*p* < 0.05 vs. wild‐type in respective treatment, two‐way ANOVA, Tukey's post hoc test.

IL‐18 is one of the cytokines increased in eWAT and iWAT from WT mice after prolonged fasting. To check whether such an IL‐18 increase is important for fat pad loss induced by prolonged fasting, we challenged the IL‐18^−/−^ mice to a 24‐h fasting. IL‐18^−/−^ mice fasted showed a reduced body weight and visceral adiposity index akin to WT mice (data not shown). IL‐18^−/−^ also diminishes their eWAT and iWAT weight (Figure 7A,D) and adipocyte area (Figure 7B,C; 7E‐F) in the same extent seen in WT mice. FFA serum levels did not alter in IL‐18^−/−^ mice after prolonged fasting, different from the increase observed in WT mice (Figure 7G). Interestingly, basal TNF expression in eWAT (Figure 7H) and iWAT (Figure 7I) from IL‐18^−/−^ mice was higher than in WT mice, with no change after fasting. Thus, it appears that the adipose tissue loss observed in IL‐18^−/−^ mice after prolonged fasting is likely due to their elevated basal TNF expression.

### 3.5 TNFR1 and IL‐18 Expression in Subcutaneous WAT Correlates With Lipase Expressions in Humans With Obesity

**FIGURE 8:**
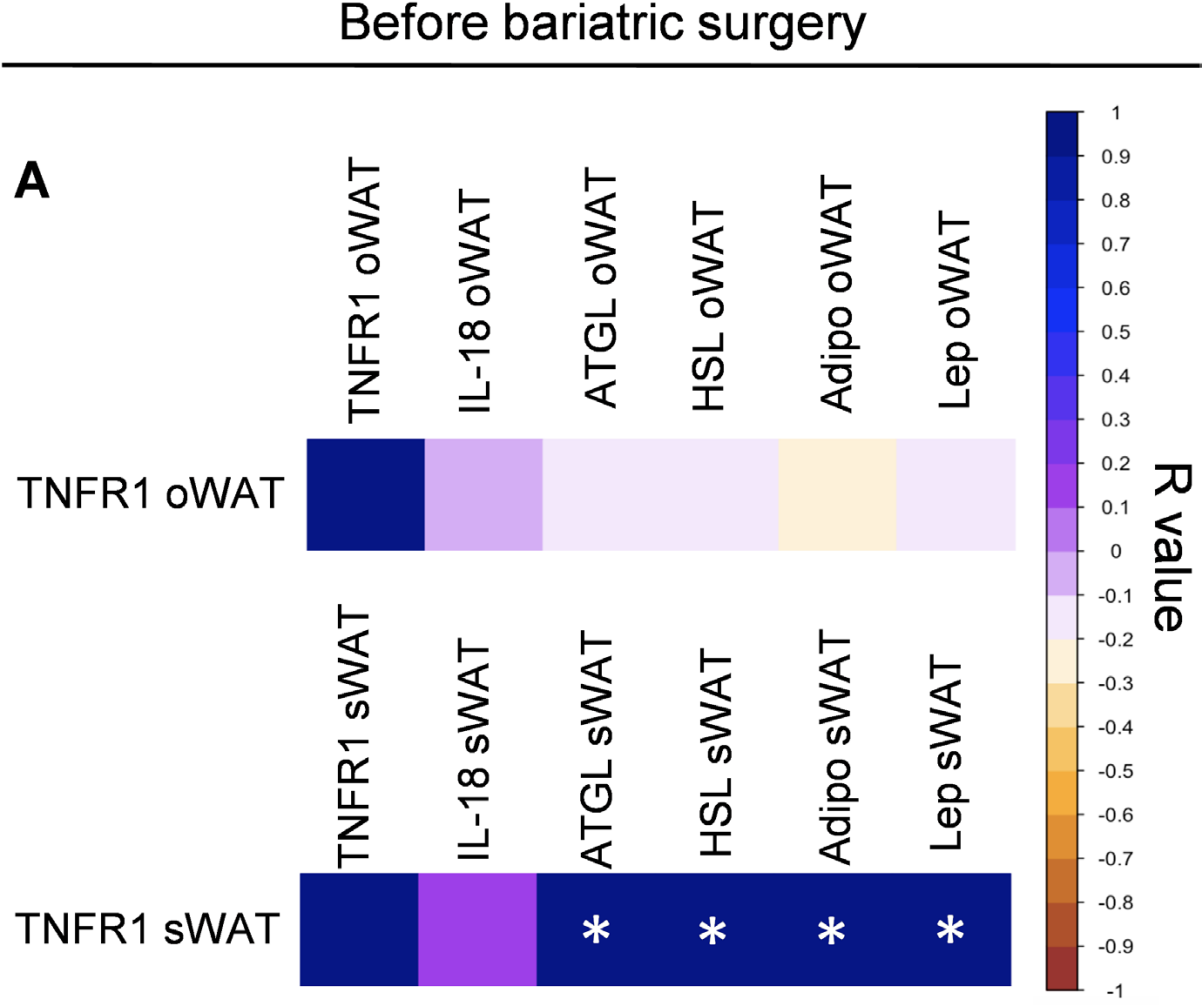
TNFR1 mRNA is positively associated with expression of ATGL, HSL Adiponectin and Leptin transcripts in sWAT but not in oWAT from subjects with severe obesity. Spearman correlation matrix of TNFR1 expression with IL‐18, lipases, and cytokines in oWAT and sWAT. *n* = 53, *p* < 0.05.

Our observations in mice led us to explore whether TNF also contributes to fat loss under other conditions of low energy availability, specifically in humans undergoing bariatric surgery. Therefore, we aimed to determine if TNF signaling is associated with adipose tissue reduction following bariatric surgery in individuals with obesity. For this purpose, we took a robust dataset from 53 patients with obesity who underwent bariatric surgery. The expression of TNFR1 in omental and subcutaneous adipose tissue was analyzed and found not to be correlated with BMI (*r* = 0.2510, *p* = 0.072) or body fat mass (*r* = 0.1714, *p* = 0.224). However, TNFR1 expression in subcutaneous adipose tissue strongly correlates with the expression of ATGL, HSL, leptin, and adiponectin (Figure 8). TNFR1 expression in oWAT or sWAT before bariatric surgery does not correlate with BMI, body weight, weight loss, and body fat loss 1 year after bariatric surgery (data not shown). No statistical correlation was observed between TNFR1 in the oWAT and sWAT for any body fat compartment, that is, android or gynoid fat, and markers of glucose metabolism (data not shown).

We also analyzed the IL‐18 expression in oWAT and sWAT from patients with severe obesity. We did not find any correlation between IL‐18 and BMI (r = −0.2695, *p* = 0.088) or body fat mass (r = −0.2203, *p* = 0.166). On the other hand, the expression of IL‐18 in omental adipose tissue strongly correlates with the expression of ATGL, HSL, leptin, and adiponectin Figure S5. IL‐18 expression in sWAT also correlates with ATGL, adiponectin, and leptin Figure S5. The changes in body composition, body weight, and fat mass loss 1 year after bariatric surgery did not correlate with the expression of IL‐18 in omental or subcutaneous adipose tissue (data not shown). No other correlation was found between the expression of IL‐18 in oWAT and sWAT with the circulating glucose, insulin, or HbA1c% (data not shown). These observations suggest that TNF and IL‐18 signaling seem to be related to adipose tissue lipases but not correlated with weight or body fat loss induced by bariatric surgery.

## 4 Discussion

Adipose tissue is a dynamic organ capable of remodeling according to energy status [1]. During fasting or calorie restriction (voluntary food intake reduction or due to bariatric surgery), a lower energy supply triggers an increase in sympathetic tone and *β*3‐AR content in white adipose tissue that, in turn, may increase the cleavage of TG stored, releasing FFA and glycerol, providing energy substrate for the organism [27]. In the last decade, a body of evidence also displayed that those inflammatory mediators favor proper fat pad loss [4, 5, 6, 8, 11]. Herein, we investigated the role of TNF and IL‐18 signaling on adipocyte shrinking and adipose tissue remodeling during a low‐energy challenge. We showed that TNF signaling is essential for visceral and subcutaneous adipose tissue reduction induced by prolonged fasting in mice. The TNF pathway seems to influence the lipolytic signaling and the content of other cytokines in adipose tissue in vivo. Interestingly, we have shown that TNFR1 expression in adipose tissue from individuals with obesity correlates positively with adipocyte lipases. On the other hand, despite IL‐18 being elevated in adipose tissue from mice upon prolonged fasting and its expression being correlated with adipose tissue lipases in humans, this cytokine does not participate in adipose tissue reduction induced by prolonged fasting (in lean mice) or by bariatric surgery in humans. These data support our hypothesis of the cytokine‐specific effect of TNF/TNFR1 on fat pad loss caused by low energy availability.

Using different experimental strategies, including TNFR1 knockout mice and pharmacological approaches, we showed that mice subjected to prolonged fasting have a blunted fat pad loss in the absence of TNF signaling. Such resistance to prolonged fasting‐induced fat pad shrinkage was also observed in mice fasted for an even longer period (48 h). The inhibition of TNF signaling with infliximab, a drug that prevents the TNF interaction with its receptors, TNFR1 and TNFR2, confirms the direct effect of TNF in controlling adipose tissue remodeling. Our analysis of the available snRNAseq dataset [20] showed that TNFR1 has higher expression in murine and human adipocytes, which agrees with experimental data demonstrating that the signaling of TNF by TNFR1 is responsible for its main functions in WAT, including lipolysis. Altogether, these data suggest that TNF may regulate fat pad loss induced by prolonged fasting through TNFR1.

Fat mobilization from adipocytes is the central core of adipose tissue remodeling induced by fasting [27]. Prolonged fasting increased FFA levels in both control and TNFR1 knockout mice. We attribute this effect to lipid mobilization from unassessed fat depots (such as brown or mesenteric adipose tissue) or to altered FFA uptake by peripheral tissues. These processes, which are modulated by TNF signaling, may contribute to the circulating FFA pool even in the absence of detectable fat mass loss. Nevertheless, only control mice displayed increased expression of ATGL and HSL in iWAT. These data are consistent with higher expression of TNFR1 in iWAT compared with eWAT. Moreover, clusters of adipocytes highly expressing TNFR1 are lipolytic (*mad3*), as evidenced by analyzing the available snRNAseq dataset [20]. Indeed, we did not find a statistical difference between the lipase expression in the 48‐h fasting, which is consistent with the literature that shows that prolonged fasting does not alter the expression of lipases but induces WAT remodeling as we observed. Thus, TNF/TNFR1 is a possible pathway that modulates lipolysis under prolonged fasting stimulus, especially in the subcutaneous adipose tissue. Previously, in vitro data showed the lipolytic effect of TNF [13, 15, 16, 29, 30] mainly by modulating the lipolytic enzymes [31]. We demonstrate in vivo the significance of this mechanism, especially in iWAT during prolonged fasting.

Sympathetic activation is a key regulator of lipolysis and adipose tissue remodeling during fasting, as it enhances sympathetic tone and *β*3‐AR signaling in white adipose tissue [2]. To determine whether TNFR1 deficiency influences sympathetic activity, we quantified tyrosine hydroxylase in WAT as a marker of sympathetic innervation. Notably, eWAT from TNFR1^−/−^ mice exhibited higher tyrosine hydroxylase levels than wild‐type controls. However, prolonged fasting did not alter tyrosine hydroxylase content in eWAT from either genotype, whereas iWAT from both strains showed an increase. Indeed, during fasting, the sympathetic tone in iWAT is higher than eWAT [32]. Taken together, these findings suggest that TNFR1 deletion does not compromise sympathetic innervation. To further assess the role of TNFR1 in *β*3‐adrenergic receptor regulation, we examined *β*3‐AR protein levels in white adipose tissue. Prolonged fasting upregulated *β*3‐AR in epididymal and inguinal WAT; however, this response was abolished in the subcutaneous depot of TNFR1^−/−^ mice. These findings suggest that TNFR1 plays a role in modulating lipolysis in subcutaneous adipose tissue by regulating *β*3‐AR expression in iWAT.

Lipolysis varies significantly depending on the anatomical location of WAT. Subcutaneous WAT expresses higher levels of the anti‐lipolytic α2‐adrenergic receptor compared to visceral WAT [33, 34]. In contrast, visceral WAT displays greater expression of *β*3‐AR and is therefore more sensitive to catecholamines [35, 36]. Based on these differences, we suggest that infliximab was unable to block lipolysis in eWAT, and that higher doses of the anti‐TNF might be required for this depot. It is also important to emphasize that sympathetic nerve organization differs between depots: visceral adipose tissue exhibits a more amorphous sympathetic nerve structure, whereas subcutaneous WAT is organized into discrete lobules [37]. Moreover, during prolonged fasting, sympathetic nerve activity is greater in subcutaneous WAT than in visceral WAT, which is consistent with our findings regarding TH fluorescence intensity. These depot‐specific differences in lipolysis may also be influenced by their anatomical proximity to distinct organs: visceral WAT is located near the portal vein, directing FFAs and glycerol generated from lipolysis to the liver, where they serve as alternative substrates to glucose, while subcutaneous WAT is closer to skeletal muscle, preferentially supplying FFAs and glycerol to that tissue. These findings suggest that while eWAT is largely regulated by sympathetic nerve activity, the TNF/TNFR1 pathway plays a more critical role in the remodeling of iWAT, highlighting distinct regulatory mechanisms for each fat depot. Together, these observations support our proposal that TNF/TNFR1 is an important pathway regulating lipolysis and iWAT remodeling during prolonged fasting.

Based on these findings, we opted to utilize the selective *β*3‐AR agonist CL316,243, which is widely employed to mimic sympathetic signaling and induce lipolysis in white adipose tissue [39]. Using ex vivo and in vivo approaches, we observed that CL316,243 treatment induced notable fat mobilization and adipocyte shrinkage. Conversely, the absence of TNF signaling attenuated lipolysis, fat pad loss, and cytokine expression in WAT stimulated by the *β*3‐AR agonist. Our data suggest that, under steady‐state conditions, TNF signaling plays a supportive role in lipolysis, facilitating proper sympathetic signaling in adipocytes and promoting physiological fat pad remodeling during prolonged fasting. Notably, in obesity, elevated TNF levels in WAT reduce *β*3‐AR expression in adipose tissue, leading to catecholamine resistance in WAT [40]. These observations, combined with our findings, support the notion that TNF signaling in white adipose tissue is essential for adipose tissue remodeling according to energy status.

Mimicry of adrenergic signaling in WAT by CL316,243 treatment also induces an inflammatory response in the tissue with an increase in monocyte infiltrate, neutrophil recruitment, and cytokine expression [8, 9, 10]. Akin to this, mice subjected to prolonged fasting had increased TNF, IL‐6, and IL‐10 levels in eWAT [6]. Herein, we confirm that wild‐type mice treated with CL316,243 showed higher expression of TNF and IL‐6 in subcutaneous and visceral adipose tissues. In contrast, in the absence of TNF signaling, there was no increase in the expression of cytokines in WAT from mice fasted or treated with CL316,243, confirming the physiological relevance of TNF signaling in modulating the expression of other cytokines [41, 42], which in turn may have an accessory effect on fat pad loss stimulated by prolonged fasting.

Fasting or pharmacologic adrenergic activation rapidly increases the immune cell content of adipose tissue [4, 6, 8, 9, 10, 11]. Following an overnight fast, the macrophage content of adipose tissue increases by around 20% [4]. Despite 12‐h fast not causing any change in adipose tissue loss, a higher influx of neutrophils and TNF‐*α* content is seen in adipose tissue, indicating an anticipated inflammatory response supporting fat remodeling [11]. Activation of lipolysis by a *β*3‐adrenergic agonist also increases by 3‐fold the macrophage content in adipose tissue within 24 h [4]. Interestingly, we also found that prolonged fasting increased the population of TIM4+ resident macrophages, an effect blunted in the absence of TNF signaling [43]. Demonstrated that those resident macrophages are necessary for adipose tissue formation during development and support lipid storage in adipocytes when energy is abundant [44]. Herein, we suggest that TIM4+ resident macrophages may also be involved in controlling fat reduction in subcutaneous adipose tissue under prolonged fasting conditions and this effect may involve TNF signaling.

Given the importance of TNF/TNFR1 signaling in adipose tissue remodeling observed in animal models, we sought to determine whether this pathway also influences fat pad loss in humans under conditions of reduced energy availability, such as after bariatric surgery. Bariatric surgery is a weight‐loss strategy designed to reduce caloric intake by restricting food consumption or altering nutrient absorption. This reduction in energy availability promotes increased lipolysis in WAT, ultimately leading to fat pad loss [45, 46]. Firstly, we found a strong correlation between lipolytic enzymes and TNFR1 expression in the subcutaneous adipose tissue of subjects with severe obesity. Although obtained in a different context from that investigated in mice, the data from our human cohort are consistent with our hypothesis that TNF regulates lipolysis. Consistent with these findings, treating TNF in primary adipocytes from humans increases lipolysis by NFκB and MAPK [15, 30]. Accordingly, we showed a higher correlation between TNFR1 expression and the lipases in subcutaneous adipose tissue. These data confirm that TNF signaling may have a supportive lipolytic effect in human adipocytes. Further, we speculate whether TNFR1 expression could be related to the weight and fat loss rate induced by bariatric surgery. Previous data show a switch in macrophages M1 to M2 phenotype in WAT three months after bariatric surgery, thereby decreasing markers of inflammation [47, 48]. However, TNF expression increases in adipose tissue of individuals 1 year after bariatric surgery, although subcutaneous adipose tissue inflammation is significantly reduced [49]. Our research did not identify any correlation between pre‐surgery TNFR1 expression in WAT and one‐year post‐surgery body weight or fat loss. This lack of association may be due to the time point evaluated, highlighting the crucial need for further analysis. We recommend matching the WAT samples collected 1 year after surgery with the surgery outcomes to better understand this association

We systematically showed here and previously [11] that some other cytokines than TNF are increased during fasting in mice, especially IL‐18. Indeed, IL‐18 seems to be related to fat mobilization since patients with lipodystrophy treated with acipimox and insulin have reduced plasma concentrations of IL‐18 [50]. IL‐18 overexpression was also related to cachexia [18]. On the other hand, IL‐18 cytokine may control adipose tissue expansion since its absence determines spontaneous obesity in mice [51, 52]. Thus, we also chose to check whether IL‐18 may have a role in fat pad loss induced by 24‐h fasting. Contrary to our hypothesis, IL‐18^−/−^ mice responded to prolonged fasting similarly to WT mice. We attribute these results to the observed increased expression of TNF in IL‐18^−/−^ mice. Therefore, our findings in the IL‐18^−/−^ mice serve as a positive control, which reinforces our hypothesis regarding the crucial role of the TNF/TNFR1 pathway in fat pad loss during prolonged fasting. Moreover, despite IL‐18 being correlated with the expression of lipases in adipose tissue in individuals with obesity, the expression of this cytokine did not correlate with markers related to weight loss and body fat 1 year after bariatric surgery. In this sense, we unveil that IL‐18 seems not essential for WAT remodeling induced by prolonged fasting, despite our clinical finding supporting the idea that in human obesity, IL‐18 expression is positively related to the lipolytic enzymes in adipose tissue.

Inflammation is a physiological response traditionally associated with defense against infections and the maintenance of systemic homeostasis [53]. More recently, we and others have proposed that inflammation also plays a pivotal role in regulating adipose tissue remodeling [6, 42, 52, 54, 55, 56, 57, 58, 59]. Indeed, under conditions of low energy availability, the inflammatory response contributes significantly to fat pad reduction, promoting the release of energy substrates and supporting systemic energy balance [4, 6, 56]. It is recognized that following more than 16‐h fasting period, mice exhibit an acute stress response aimed at restoring homeostasis, which can affect the processes involved in adipose tissue loss through the activation of hormonal and inflammatory pathways [60]. Accordingly, our observations from 24‐ or 48‐h fasting periods illustrate that reductions in adipose tissue are attributable to both physiological adaptations and stress‐related responses.

### 4.1 Study Limitations and Future Perspectives

We acknowledge the primary limitations of this work. Firstly, our mechanistic conclusions are primarily drawn from the male C57Bl/6J mouse model. Another limitation of our study is that we did not investigate the effects of fasting in female mice. This analysis would require controlling for the estrous cycle, as fasting responses are known to be strongly influenced by hormonal fluctuations [61]. Therefore, additional studies are needed to address this aspect and determine whether similar mechanisms occur in females.

Secondly, our inability to use an adipocyte‐specific TNFR1 knockout model limits our ability to pinpoint cell‐type‐specific effects, though our systemic and ex vivo approaches strongly point to a key role in the fat pad. Furthermore, we focus on TNFR1 due to its predominant expression in adipocytes, but we acknowledge that TNFR2 may also influence adipose tissue remodeling.

Regarding our clinical data, the cohort presents specific limitations, including the predominance of women [62] and the challenge of obtaining tissue samples from lean individuals or those 1 year after bariatric surgery. Despite these constraints, our clinical data are consistent, as we have a sample of 53 individuals, and the correlation between TNFR1 expression in sWAT and lipase expression is strong. This molecular finding is further supported by evidence that, beyond the quantitative dominance of TNFR1 expression, our clinical data demonstrate the molecular functional correlation of this pathway in human adipocytes. This finding supports the hypothesis that the TNF/TNFR1 axis is intrinsically linked to the lipolytic capacity of the human adipocyte. We acknowledge that the lack of correlation with complex, long‐term clinical outcomes (e.g., one‐year weight loss) reflects the multifactorial nature of post‐surgery results and does not negate this strong molecular association. Translational confirmation of this mechanism in female mice and different human cohorts remains essential for determining the broader physiological relevance of our findings.

### 4.2 Conclusions

In this study, we investigated whether TNF or IL‐18 independently regulates fat loss during prolonged fasting in male mice. We demonstrated that TNF signaling is essential for effective fat mobilization, supporting both the expression of lipolytic enzymes and downstream sympathetic signaling induced by prolonged fasting in male mice. Furthermore, TNF exerts pleiotropic effects by modulating other cytokines, including IL‐6, IL‐18, and IL‐10, and by influencing the activity of TIM4^+^ resident macrophages. Although IL‐18 expression increases in WAT during prolonged fasting and correlates with lipolytic markers in humans, IL‐18 deficiency in mice does not impair fat loss, indicating that IL‐18 is not required for prolonged fasting‐induced adipose tissue remodeling. Together, our findings reveal a cytokine‐specific mechanism through which inflammation regulates physiological lipolysis, with TNF playing a central role. Human expression data support the translational relevance of these receptors, but future studies are required to confirm this mechanism in female mice and in human cohorts. Still, this work has significant translational implications for understanding the inflammatory regulation of energy metabolism and may inform therapeutic strategies to optimize adipose tissue function during obesity treatment and weight loss interventions.

## Author Contributions

A.C.C.O., G.M., A.V.M.F.: conceptualization and study design; A.C.C.O., A.M.B, M.L.S., L.S.B.L., T.S.R., M.C.O., C.R., E.L.G., A.G.O., G.M.: investigation; M.M.T., K.C., G.M., A.V.M.F.: funding acquisition and resources; A.C.C.O., M.C.O., M.M.T., K.C., G.M., A.V.M.F.: Writing, original draft, review and editing.

## Funding

This work was supported by “Coordenação de Aperfeiçoameto de Pessoal de Nível Superior” (CAPES) and “Comité français d’Evaluation de la Coopération Universitaire et Scientifique avec le Brésil” (COFECUB) grant no 88881.879204/2023‐01, “Conselho Nacional de Desenvolvimento Científico e Tecnológico” (CNPq), Fundação de Amparo à Pesquisa de Minas Gerais (FAPEMIG) and Instituto Nacional em Ciência e Biotecnologia (INCT) Nanobiofar, grant 406792/2022‐4.

## Conflicts of Interest

The authors declare no conflicts of interest.

## Supporting information


**Data S1:** Supporting Information.

## Data Availability

Included in article.
