# Supplementary material for: TNF/TNFR1 is a Key Regulator of Prolonged Fasting‐Induced Decrease in Adipose Tissue
Source: FASEB J. 2026 Jan 8;40(1):e71404. doi: 10.1096/fj.202501928RR (PMC12784137; doi:10.1096/fj.202501928RR)
Supplement: Supplementary file 1 — Data S1: Supporting Information. [file FSB2-40-e71404-s001.pdf]

Supplementary Table 1 - Anthropometric and biochemical characteristics of patients with severe obesity before bariatric surgery.

Variables	Patients with severe obesity
**Female (%)**	81
**Age (years)**	49.6±1.5
**Body Weight (Kg)**	126.6 ± 3.2
**BMI (kg/m^2)**	46.4 ± 1.2
**Fat mass (Kg)**	60.3 ± 2.0
**Muscle Mass (Kg)**	62.4 ± 2.1
**Fasting Glucose (mmol/l)**	6.3 ± 0.2
**Fasting Insulin(μUI/l)**	21.2 ± 2.2
**HbA1c (%)**	6.05 ± 0.1

Supplementary Table 2 - List of primer sequences used for qPCR analysis in mouse and human samples.

Gene	Foward	Reverse
**18S**	CGATGCTCTTAGCTGAGTGT	GGTCCAAGAATTTCACCTCT
**RPLP0**	ACAGGGCGACCTGGAAGT	GGATCTGCTGCATCTGCTT
**LIPE**	AGCGCTGGAGGAGTGTTTT	CCGCTCTCCAGTTGAACC
**PNPLA2**	GAGCTTCGCGTCACCAAC	CACATCTCTCGGAGGACCA
**TNF**	GTAGCCCACGTCGTAGCAAAC	AGTTGGTTGTCTTTGAGATCCATG
**IL-6**	GCTACCAAACTGGATATAATCAGG	CCAGGTAGCTATGGTACTCCAGAA
**IL-10**	CCCTGCTTGCACATCTCTC	CCCTGCTTGCACATCTCTC
**IL-18**	AACCTCCAGCATCAGGACAAAG	TTTCCTTGAAGTTGACGCAAGA
**18s human**	CGATGCTCTTAGCTGAGTGT	GGTCCAAGAATTTCACCTCT
**RPLP0 human**	ACAGGGCGACCTGGAAGT	GGATCTGCTGCATCTGCTT
**TNFR1 human**	TCCTTCACCGCTTCAGAAA	GGGATAAAAGGCAAAGACCAA
**LIPE human**	CGCTGGAGGAGTGCTTCTT	TTCGTTCCCCTGTTGAGC
**PNPLA2 human**	CTCCACCAACATCCACGAG	CCCTGCTTGCACATCTCTC
**Leptin human**	TTGTCACCAGGATCAATGACA	GTCCAAACCGGTGACTTTCT
**Adiponectin human**	AGAGATGGCACCCCTGGT	CACCGATGTCTCCCTTAGGA
**IL-18 human**	GCTTCCTCTCGCAACAAACT	TGATGCAATTGTCTTCTACTGGTT

Supplementary Table 3 - Average gene expression of TNFR1 and TNFR2 across different adipocyte clusters identified by single-nucleus RNA sequencing (snRNAseq).

**Gene**	**Average gene expression**	**Adipocyte clusters**	**Mean**	**Standard deviation**	***p-value***
TNFR1	0.161	mAd1	0.161	0.643	6.11e-77
TNFR2	0.071		0.071	0.384	
TNFR1	0.165	mAd2	0.165	0.585	0.239
TNFR2	0.153		0.153	0.528	
TNFR1	0.531	mAd3	0.531	1.40	3.34e-23
TNFR2	0.113		0.113	0.554	
TNFR1	0.191	mAd4	0.191	0.570	1.12e-07
TNFR2	0.141		0.141	0.440	
TNFR1	0.217	mAd5	0.217	0.563	7.56e-14
TNFR2	0.101		0.101	0.366	
TNFR1	0.429	mAd6	0.429	0.839	5.32e-05
TNFR2	0.247		0.247	0.661	

Supplementary Figure 1

A **Mice adipocytes**

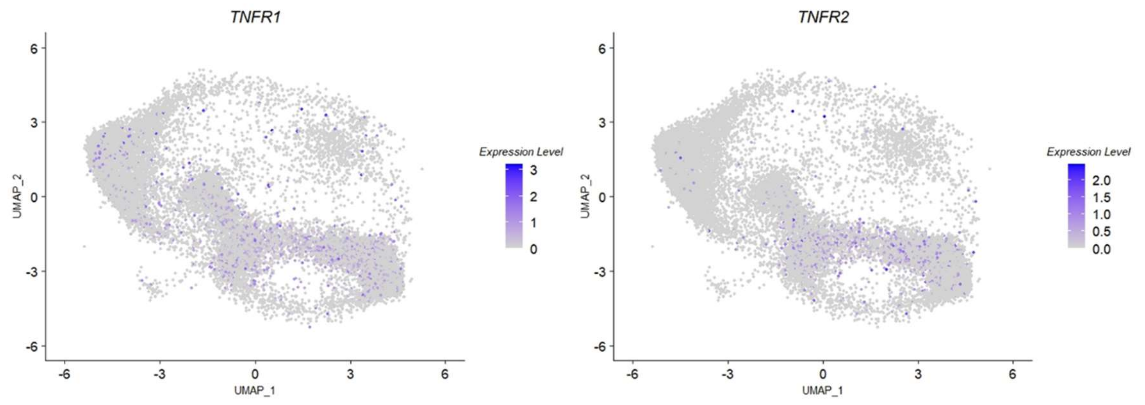

B **Human adipocyte**

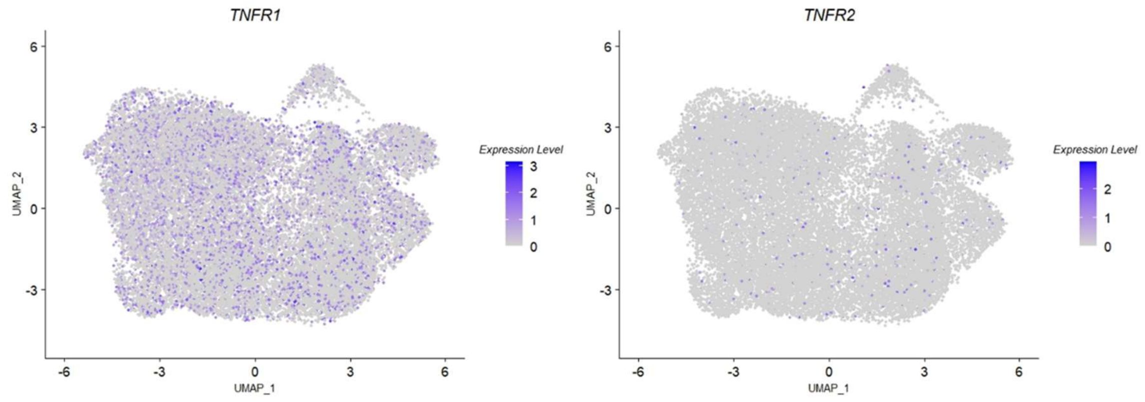

Mice and human adipocytes express more TNFR1 than TNFR2. UMAP plots displaying TNFR1 and TNFR2 expression in mice (A) and human (B) adipocytes. The adipocytes were identified using single-nucleus RNA sequencing (snRNAseq) on cells from pooled subcutaneous and visceral depots. The color scale bar represents the normalized expression level for the respective gene clusters shown in the UMAP plot. The gradient indicates the expression range, with shades ranging from gray (low expression) to blue (high expression). Data were obtained from Emont et al. (2022; GSE176171).

Supplementary Figure 2

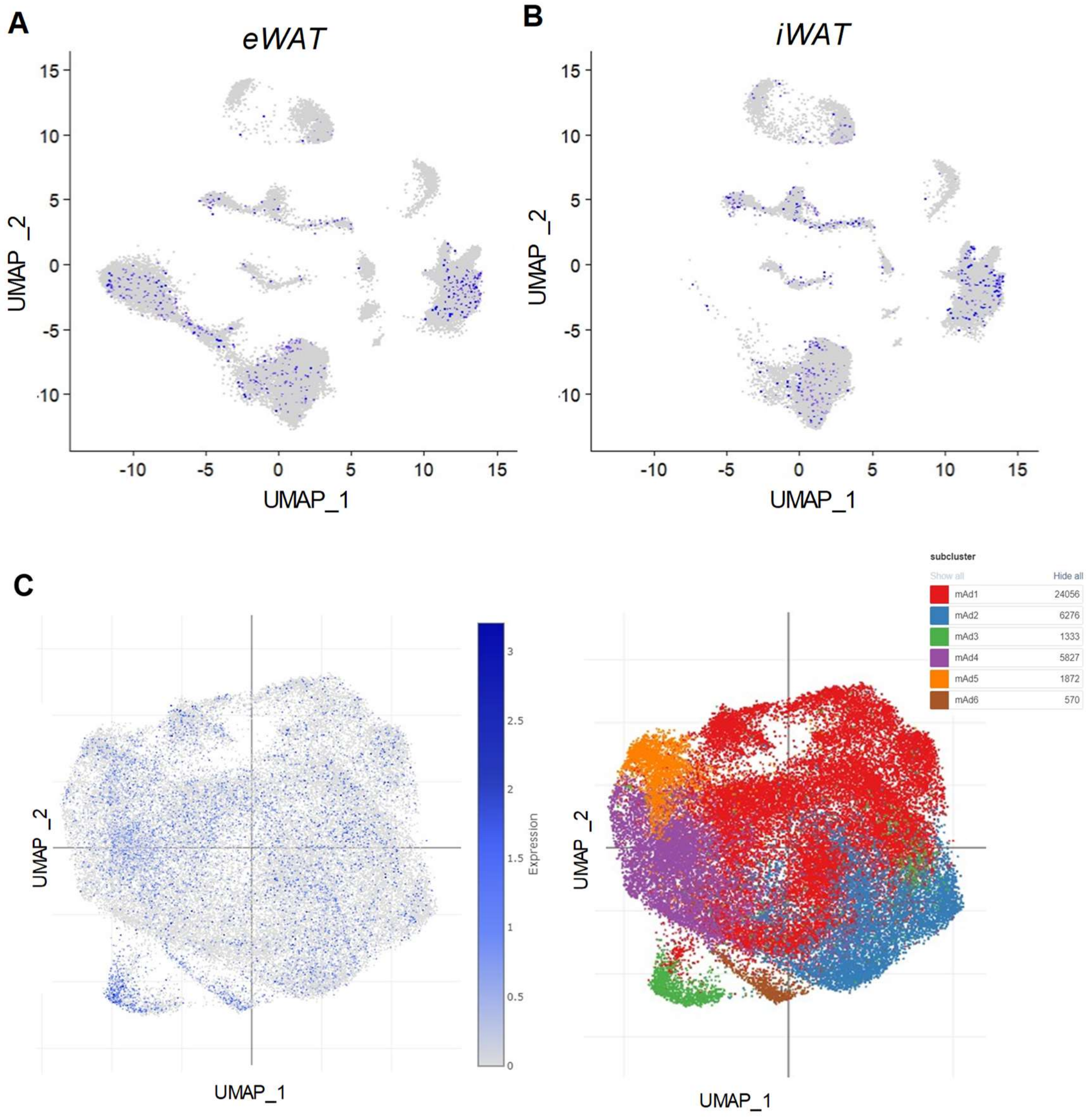

The expression of TNFR1 is not only specific to fat depot but also directly relates to the subpopulation of adipocytes with lipolytic characteristics. (A) The UMAP plot of cells (GSE176171) in eWAT and the expression of TNFR1. (B) The UMAP plot of cells (GSE176171) in iWAT and the expression of TNFR1. (C) The UMAP plot of cell types (GSE176171) and TNFR1 expression in adipocytes subclusters. This detailed data is available at Emont et al. 2022 (doi: 10.1038/s41586-022-04518-2).

Supplementary Figure 3

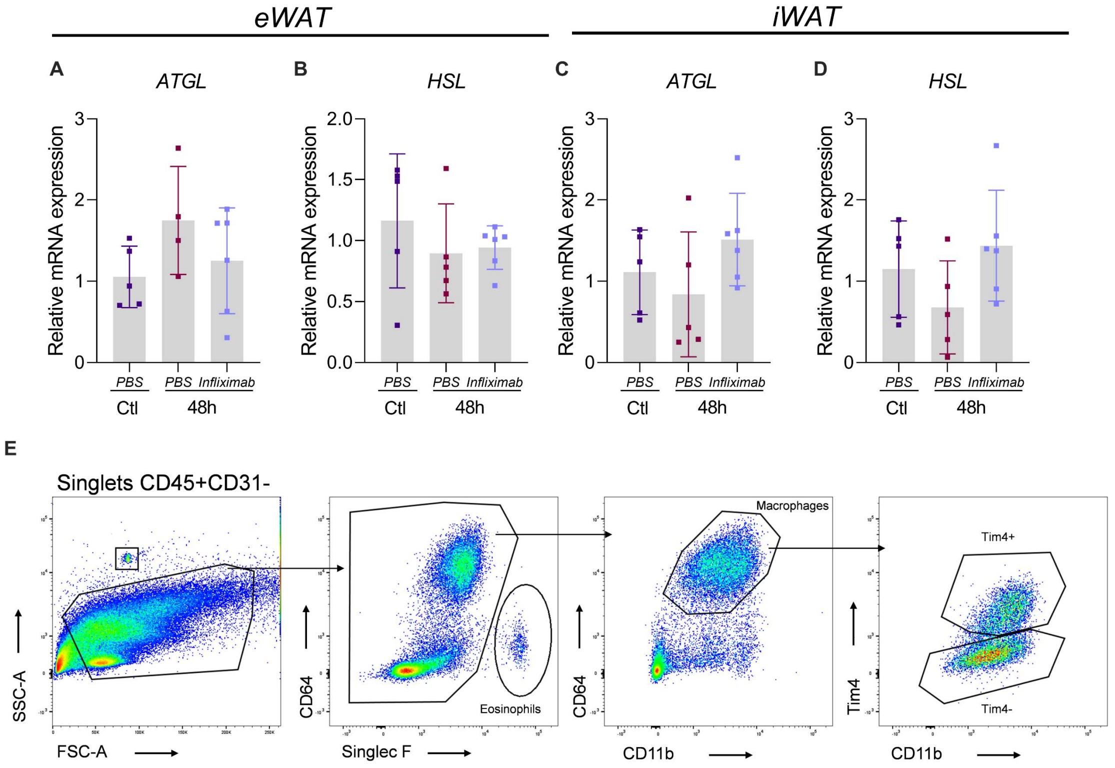

Anti-TNF treatment did not alter the expression of lipases in WAT. C57BL/6 mice were treated with PBS or infliximab and submitted (48h) or not (ctl) to 48h fasting. ATGL and HSL expression in (A and B) eWAT and (C and D) iWAT, n=4-8, *p< 0.05 vs. respective ctl group, #*p< 0.05 vs. wild-type in respective treatment, two-way ANOVA, Tukey's posthoc test. (E) The sequential gating strategy used to identify and analyze macrophage populations from mouse adipose tissue via flow cytometry.

Supplementary Figure 4

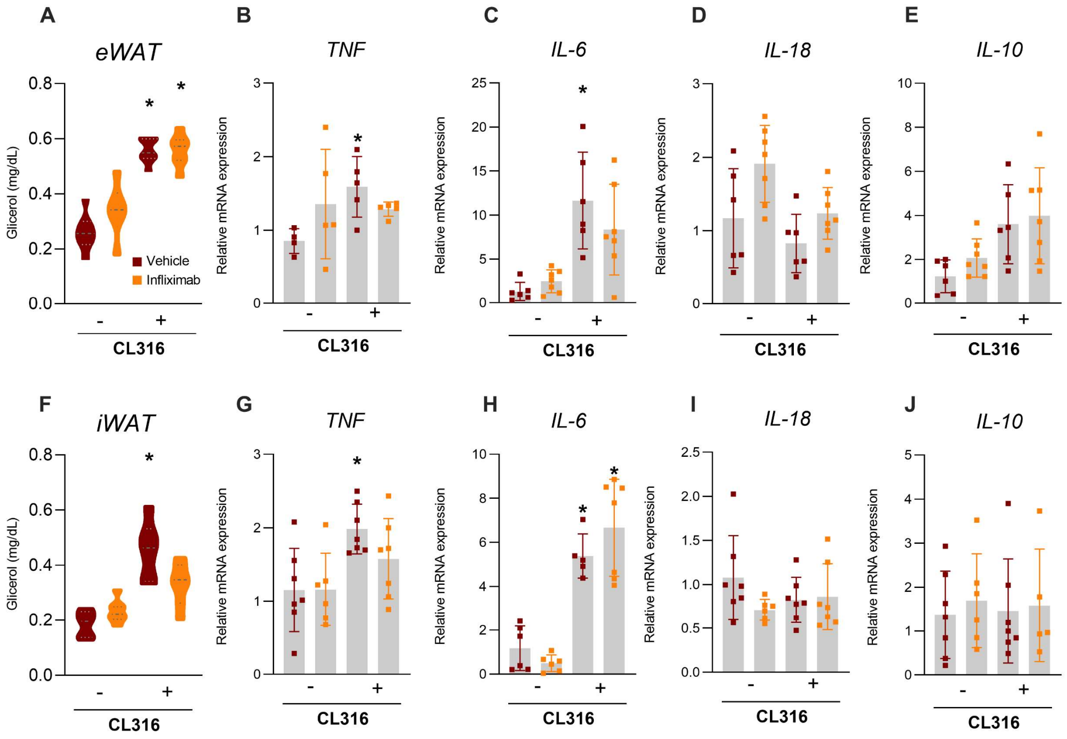

The TNF pathway is actively involved in the lipolysis induced by CL316,243 in iWAT but not in eWAT. Explants of eWAT and iWAT were treated with PBS or infliximab and stimulated with CL316,243 or not. (A) eWAT glycerol release, (B) TNF, (C) IL-6, (D) IL-18 and (E) IL-10 expression from eWAT. (F) eWAT glycerol release, (G) TNF, (H) IL-6, (I) IL-18 and (J) IL-10 expression from eWAT. n=6, *p< 0.05 refers to basal (-) vs. stimulated (+), and #p< 0.05 refers to the statistical difference between vehicle and infliximab, two-way ANOVA, Tukey's posthoc test.

Supplementary Figure 5

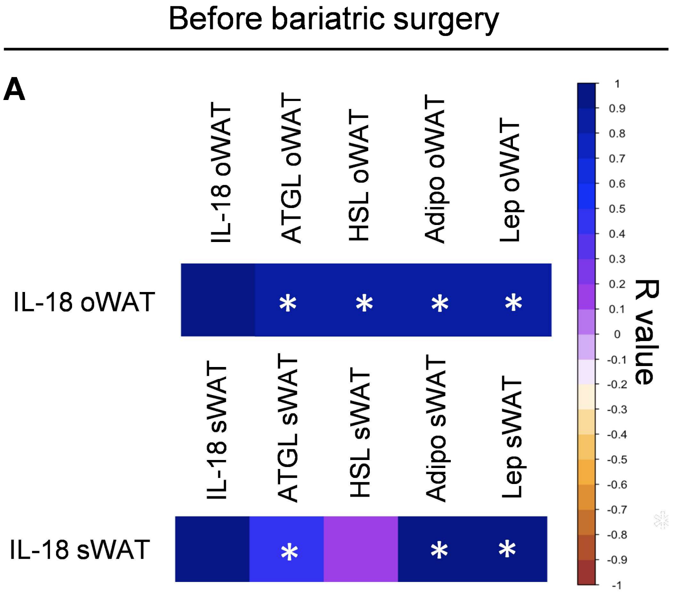

IL-18 mRNA is positively associated with expression of HSL, Adiponectin and Leptin transcripts in human adipose tissue from fasted subjects with severe obesity. (A) Spearman correlation matrix of IL-18 expression with lipases and adipocytokines in oWAT and sWAT before bariatric surgery. n= 53, *p< 0.05.
